# Cross-Reactivity Assessment of Vaccine-Derived SARS-CoV-2 T Cell Responses against BA.2.86 and JN.1

**DOI:** 10.3390/v16030473

**Published:** 2024-03-20

**Authors:** Muhammad Saqib Sohail, Syed Faraz Ahmed, Ahmed Abdul Quadeer, Matthew R. McKay

**Affiliations:** 1 Department of Electronic and Computer Engineering, The Hong Kong University of Science and Technology, Hong Kong SAR, China; mssohail@connect.ust.hk; 2 Department of Electrical and Electronic Engineering, University of Melbourne, Parkville, VIC 3010, Australia; faraz.ahmed@unimelb.edu.au; 3 Department of Microbiology and Immunology, The Peter Doherty Institute for Infection and Immunity, University of Melbourne, Melbourne, VIC 3000, Australia

**Keywords:** SARS-CoV-2, COVID-19, BA.2.86, JN.1, T cell epitopes, immune escape, mutations, vaccines

## Abstract

The SARS-CoV-2 Omicron sub-variants BA.2.86 and JN.1 contain multiple mutations in the spike protein that were not present in previous variants of concern and Omicron sub-variants. Preliminary research suggests that these variants reduce the neutralizing capability of antibodies induced by vaccines, which is particularly significant for JN.1. This raises concern as many widely deployed COVID-19 vaccines are based on the spike protein of the ancestral Wuhan strain of SARS-CoV-2. While T cell responses have been shown to be robust against previous SARS-CoV-2 variants, less is known about the impact of mutations in BA.2.86 and JN.1 on T cell responses. We evaluate the effect of mutations specific to BA.2.86 and JN.1 on experimentally determined T cell epitopes derived from the spike protein of the ancestral Wuhan strain and the spike protein of the XBB.1.5 strain that has been recommended as a booster vaccine. Our data suggest that BA.2.86 and JN.1 affect numerous T cell epitopes in spike compared to previous variants; however, the widespread loss of T cell recognition against these variants is unlikely.

## 1. Introduction

The emergence of the SARS-CoV-2 Omicron BA.2.86 variant and its closely related descendent JN.1 has raised concerns regarding their ability to evade immune responses. Despite the large number of mutations in BA.2.86 and its high receptor binding affinity, it failed to achieve global dominance due to the absence of substantial humoral immune escape and growth advantages compared to prevailing variants [1,2,3,4]. In contrast, the JN.1 variant, with a single additional mutation (L455S) in the spike of its BA.2.86 ancestor, has rapidly become the predominant strain worldwide (global prevalence > 72%, based on sequence data submitted to GISAID as of 21 January 2024 [5]). This dominance is seemingly driven by its significantly enhanced immune escape capabilities. Experimental evidence demonstrated reduced neutralization titers in breakthrough infection sera from recent variants (XBB.1.5 and EG.5.1) [6,7] and robust resistance to monovalent XBB.1.5 vaccine sera for JN.1 compared to BA.2.86 [7]. Thus, JN.1 appears to be the most immune-evasive variant observed to date. However, the impact of mutations present in the BA.2.86 and JN.1 variants on T cell responses against SARS-CoV-2 remains largely unknown.

Understanding T cell responses is particularly important for SARS-CoV-2. In vaccinated individuals, rapid cellular immune responses (both CD4+ and CD8+) were observed after breakthrough infections [8,9], correlating with milder disease outcomes [9]. Another study correlated early-activated T cell responses with lower peak viral loads and faster viral clearance [10,11]. T cells have also been found to play a distinct protective role during acute COVID-19 [12]. 

Here, we perform an in-silico investigation of the cross-reactivity of COVID-19 vaccine-induced T cell responses against the BA.2.86 and JN.1 variants. Our approach quantifies the sequence conservation of experimentally determined SARS-CoV-2 epitopes within BA.2.86 and JN.1 and assesses the potential for T cell escape through the use of human leukocyte antigen (HLA)–peptide binding tools. 

## 2. Materials and Methods

T cell epitope data were downloaded from the immune epitope database (IEDB) [13] (accessed on 8 September 2023). These epitopes have been reported to be targeted by individuals from diverse populations/cohorts following natural infection or vaccination, thereby providing a broad picture of the impact of mutations in new variants on T cell responses. We identified 565 CD4+ and 377 CD8+ S-specific experimentally determined T cell epitopes of SARS-CoV-2 that mapped onto the ancestral (Wuhan) strain (NCBI GenBank accession ID: NC_045512.2). The variant-defining mutations for the SARS-CoV-2 variants Alpha, Beta, Delta, Omicron BA.1, BA.2, BA.5, XBB.1.5, and EG.5.1 were obtained from CoVariants (https://covariants.org) [14] accessed 5 November 2023, while those of BA.2.86 and JN.1 were obtained from Outbreak.info (http://www.outbreak.info) [15] accessed 5 November 2023.

We examined all identified T cell epitopes for SARS-CoV-2 variant-defining mutations. If a variant-defining mutation occurred within a known T cell epitope, we referred to the altered sequence as an epitope mutant. To assess whether an identified epitope mutant affects binding between the associated epitope and its corresponding HLA allele, we employed established peptide–HLA binding prediction tools, netMHCIIpan4.0 [16] (for CD4+ T cell epitopes) and netMHCpan4.1 [17] (for CD8+ T cell epitopes). The predictions of these computational tools have lined up well with SARS-CoV-2 experimental studies describing T cell epitopes [18,19,20], as well as those reporting the HLA binding of specific T cell epitopes [21,22]. For all predictions, we utilized the eluted ligand percentile ranks generated by both methods that take into account peptide–MHC binding affinity as well as antigen processing and presentation. Both models were run using their default parameter settings.

## 3. Results

We first investigated the spike (S) protein, which is the antigen used in most COVID-19 vaccines. S-specific T cell responses are also known to be immunodominant upon natural infection [23]. We found 565 CD4+ and 377 CD8+ S-specific experimentally determined T cell epitopes of SARS-CoV-2 that mapped onto the ancestral (Wuhan) strain. This strain was predominantly used as the basis for synthesizing peptides used to assess T cell responses from infection or first-generation vaccines. Therefore, most SARS-CoV-2 T cell responses reported in the literature are against peptides derived from the ancestral strain.

**Figure 1:**
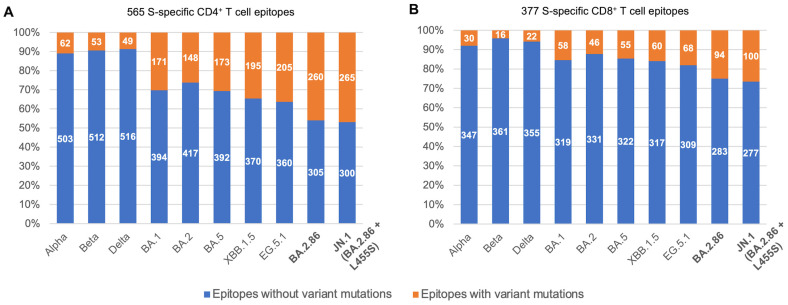
Overview of S-specific SARS-CoV-2 T cell epitopes across SARS-CoV-2 variants. (**A**,**B**) show the fractions of CD4+ and CD8+ S-specific SARS-CoV-2 T cell epitopes with and without mutations across SARS-CoV-2 variants.

Genomic screening revealed that the number of S-specific T cell epitopes affected by BA.2.86 mutations was notably larger than those affected by previous variants of concern (Figure 1A,B). For CD4+ T cells, 46% (260/565) of epitopes are affected in BA.2.86 (see Supplementary Table S1). This is a significant jump from the 30% (171/565) of CD4+ epitopes affected in the first Omicron variant (BA.1). In the case of the recently dominant JN.1 variant, which includes an additional L455S mutation within the spike of BA.2.86, the number of affected CD4+ epitopes is slightly higher at 47% (265 out of 565). More recent variants have larger numbers of epitope mutants than earlier variants, with the circulating XBB.1.5 and EG.5.1 strains having 35% (195/565) and 36% (205/565) of CD4+ epitopes affected, respectively. For CD8+ T cells, similar trends are observed, though the fraction of epitopes affected is smaller. The fraction of affected CD8+ epitopes is 25% (94/377) for BA.2.86 and 27% (100/377) for JN.1 (see Supplementary Table S2) compared with 15% (58/377) for Omicron (BA.1), 16% (60/377) for XBB.1.5, and 18% (68/377) for EG.5.1.

**Figure 2:**
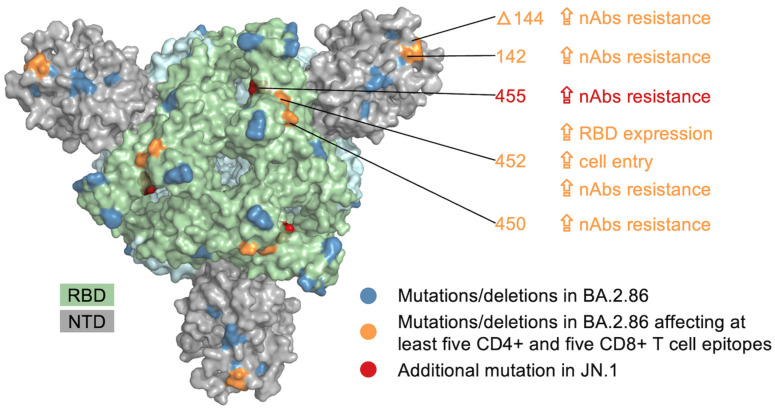
Mapping of mutations in the BA.2.86 and JN.1 variants onto the spike crystal structure. Mutations affecting numerous T cell epitopes are annotated. The additional phenotypic effects of these mutations are also noted. The crystal structure was obtained from www.rcsb.org (PDB ID: 7XIX) accessed 10 November 2023. RBD: receptor-binding domain; NTD: N-terminal domain; nAbs: neutralizing antibodies.

**Figure 3:**
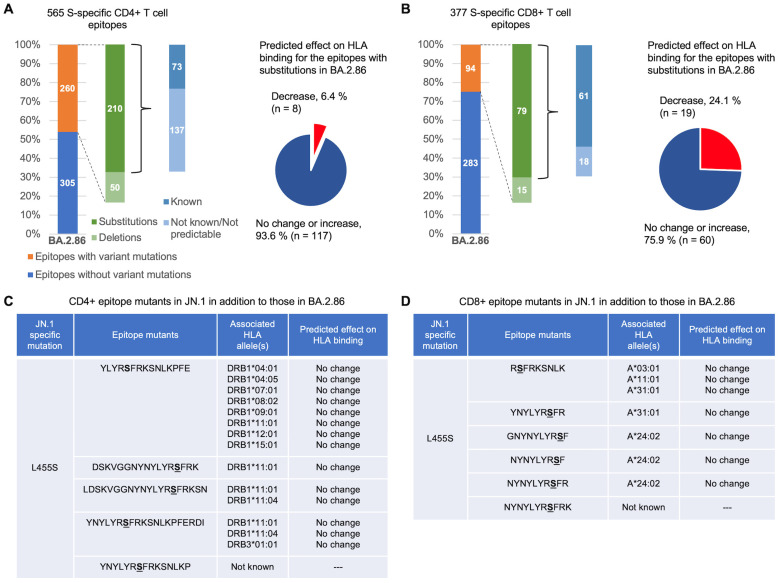
Predicted effect on the HLA binding of S-specific epitope mutants of BA.2.86 and JN.1. (**A**,**B**) show breakdowns of BA.2.86 epitopes for which HLA association is known, along with the predicted effect of Omicron BA.2.86 mutations on peptide–HLA binding. (**C**,**D**) show lists of epitopes in JN.1 (additional to those in BA.2.86) with known HLAs, along with their predicted effect on peptide–HLA binding. The complete list of CD4+ and CD8+ (mutant) epitope–HLA pairs and their predictions is provided in Supplementary Tables S3 and S4.

A single mutation can provide multiple advantages to the virus, including functional, structural, and immune escape benefits. Thus, we investigated whether BA.2.86 mutations that impact a substantial number of T cell epitopes (Figure 1A,B) had functional or structural significance. Specifically, we focused on mutations that affect a minimum of five CD4+ and CD8+ T cell epitopes each. Four BA.2.86 mutations/deletions within S met this criterion: a mutation at residue 142 and a deletion at residue 144 in the N-terminal domain (NTD), and two mutations at residues 450 and 452 in the receptor-binding domain (RBD) (see Figure 2). The existing literature indicates that these mutations are associated with increased resistance against neutralizing antibodies targeting the NTD or RBD [24,25,26,27,28]. The mutation at position 452 also provides functional advantages, including enhanced RBD expression and increased cell entry of the virus [6]. Moreover, the JN.1-specific S mutation at position 455, located in the RBD, also affects a notable number of T cell epitopes (five CD4+ and six CD8+ T cell epitopes; see Figure 3C,D). Like the other S mutations mentioned above, it increases resistance to neutralizing antibodies [6,24,27] (Figure 2). Thus, these findings suggest that specific BA.2.86 and JN.1 mutations within S have the potential to impact many known T cell epitopes while also exhibiting additional antibody escape and functional advantages. 

Some epitopes, referred to as immunodominant, have been identified to be frequently targeted in the global population. Escape from these epitopes could have widespread significance. We previously reported four S-derived CD8+ epitopes that were immunodominant across different ethnicities and geographical regions [29]. Each of these epitopes remains unmutated in BA.2.86 and JN.1. Another significant CD8+ epitope is the S-derived HLA-B15-restricted epitope _919_NQKLIANQF_927_, which has been reported to be associated with asymptomatic infection [30]. This epitope is also unmutated in both BA.2.86 and JN.1. For CD4+, a literature search revealed an S-specific CD4+ epitope, _811_KPSKRSFIEDLLFNKVTLADA_831_, that is broadly recognized in the human population (restricted by HLA-DQ5 and HLA-DP4) [31]. Another S-specific CD4+ epitope, _167_TFEYVSQPFLMDLE_180_ (restricted by DPB1 and DPB4), is also reported as immunodominant [32,33]. Both these putative immunodominant CD4+ epitopes are conserved in BA.2.86 and JN.1. Taken together, this analysis suggests that T cell targeting of immunodominant epitopes would likely be preserved with current SARS-CoV-2 vaccines based on ancestral S antigens.

To gain a better understanding of the potential impact of BA.2.86 and JN.1 mutations on T cell escape, we conducted a detailed analysis of the affected T cell epitopes. We focus first on the BA.2.86 variant. Of the epitopes affected by BA.2.86 mutations, 50/260 CD4+ and 15/94 CD8+ epitopes are lost due to deletions of one or more residues within the epitopes. T cell recognition of these epitopes is therefore likely to be abrogated in BA.2.86. The remaining affected epitopes (210/260 CD4+ and 79/94 CD8+) contain one or more amino acid mutations in BA.2.86 (Figure 3; Supplementary Tables S3 and S4). To assess whether these epitope mutants are likely to influence T cell escape, we used computational tools (see Section 2) to predict their effect on HLA binding, applying the same methodology as in [34]. HLA binding is a prerequisite for T cell recognition, and a loss of binding can facilitate T cell escape. Of the epitopes identified to carry BA.2.86 mutations, HLA allele associations are known for 73 (34.8%) CD4+ and 61 (77.2%) CD8+ T cell epitopes, resulting in 125 CD4+ and 79 CD8+ distinct (mutant) epitope–HLA pairs (Figure 3). Of these, only 8 (6.4%) of the CD4+ and 19 (24.1%) of the CD8+ (mutant) epitope–HLA pairs are predicted to have a decrease in peptide–HLA binding (Figure 3A,B) relative to the corresponding (unmutated) epitope–HLA pairs (see Supplementary Tables S3 and S4). For the JN.1 variant, the additional L455S mutation does not impact the binding of any associated epitope to its corresponding HLA allele (Figure 3C,D). For the mutations/deletions in BA.2.86 and JN.1 identified earlier that affect at least five CD4+ and five CD8+ epitopes while having additional functional advantages (Figure 2), only one of the associated affected epitopes (HLA-A*01:01-restricted _135_FCNDPFL**D**VYH_145_) is predicted to reduce HLA binding. However, all epitopes encompassing the 144 deletion are likely to abrogate T cell recognition. Overall, this analysis suggests that BA.2.86 and JN.1 mutations do not significantly affect the binding of known T cell epitopes to their associated HLA alleles.

Thus far, we have defined a mutation as any change in a variant compared to the ancestral (Wuhan) strain, which served as the basis for antigens used in initial COVID-19 vaccines. However, the use of recent COVID-19 vaccines containing antigens derived from Omicron-like strains, which are genetically more similar to BA.2.86 and JN.1 than the ancestral strain, may reduce the impact of BA.2.86 and JN.1 mutations on vaccine-induced responses even further. On 12 September 2023, the Centers for Disease Control and Prevention (CDC) of the United States (US) recommended the monovalent BNT162b2 booster vaccine, which employs an XBB.1.5 spike antigen, for everyone aged six months and older [35]. This booster has been demonstrated to elicit neutralizing antibody titers against BA.2.86 that are 8.7-fold higher compared to the levels observed prior to vaccination [36]. To predict the effect of BA.2.86 mutations on T cell responses elicited by the XBB.1.5 BNT162b2 booster, we analyzed the set of S-specific T cell epitopes (370 CD4+ and 317 CD8+) that are conserved in both the Wuhan strain and the XBB.1.5 variant. These correspond to epitopes that are conserved in XBB.1.5 and against which responses have been reported in experimental studies. Among this set of epitopes, 78.4% (290/370) of CD4+ and 86.4% (274/317) of CD8+ epitopes remain unaffected by BA.2.86 mutations. When compared with the fractions of unaffected CD4+ and CD8+ epitopes among the entire sets of epitopes (54% (305/565) for CD4+ and 75% (283/377) for CD8+), these data suggest that S-specific T cell responses elicited by the XBB.1.5-derived booster shot may be less prone to T cell escape by BA.2.86 and JN.1 compared with vaccines based on ancestral S antigens.

Our analysis has focused on T cell responses against the S protein. Responses against non-S proteins are important for COVID-19 vaccines based on whole inactivated viruses and for T cell immunity arising from natural SARS-CoV-2 infections. When extended to proteins other than S, a large majority of experimentally determined T cell epitopes (553/613 CD4+ and 1025/1094 CD8+ epitopes) remain conserved in BA.2.86 (Supplementary Figure S1A,C). ORF1ab has the highest number of epitopes, with 286/297 (96.3%) CD4+ and 811/836 (97%) CD8+ epitopes remaining conserved in BA.2.86. Within the highly expressed immunogenic N protein [37], 151/175 (86.3%) CD4+ and 97/109 (88.9%) CD8+ T cell epitopes remain conserved. Among other proteins, the structural Membrane protein (M) has the most significant fraction of mutated epitopes, with more than a quarter of both CD4+ (21/83) and CD8+ (23/92) epitopes affected by BA.2.86-specific mutations. Only a few epitopes (three CD4+ and four CD8+) outside of S are affected by deletions. Among the mutant epitope–HLA pairs in proteins other than S, only a small fraction of pairs (1/58 (1.8%) CD4+ and 10/63 (15.9%) CD8+) are predicted to have a reduction in HLA binding (Supplementary Figure S1B,D). Compared to BA.2.86, JN.1 comprises two more mutations in non-S proteins (ORF1a:R3821K and ORF7b:F19L). Epitopes comprising these mutations are predicted to maintain binding with their associated HLA alleles. Moreover, in non-S proteins, 16 immunodominant CD8+ epitopes have been identified [29]. Of these, only a single epitope, _26_FLFTWICL_34_ (located in M), harbors a BA.2.86-specific mutation (T30A). This mutation, however, is predicted to maintain binding to its associated HLA allele (HLA-A*02:01). Taken together, epitope variation within the non-S proteins of BA.2.86 is modest, and the disruption of infection-induced or vaccine-induced T cell responses toward non-S proteins is expected to be minimal.

## 4. Discussion

S-specific T cell responses against SARS-CoV-2 are important because the S protein is a dominant target of T cells and most widely deployed COVID-19 vaccines are based on a S-protein antigen. Our analysis shows that many known S-specific T cell epitopes remain conserved in BA.2.86 and JN.1. However, there is still a notable jump in the number of epitopes affected by mutations in BA.2.86 and JN.1 compared to previous variants, particularly for CD4+ epitopes. T cell responses against previous SARS-CoV-2 variants, including Omicron, have generally been shown to be preserved in different populations [18,38,39]. However, for the first Omicron subvariant BA.1 [18,39], 20% of vaccinated individuals in a US cohort were reported to experience a greater than 50% loss of T cell recognition [40]. Given that the number of T cell epitopes predicted to be affected by BA.2.86 and JN.1 mutations is 1.5 times higher than those affected by BA.1 mutations (Figure 1), a further reduction in T cell responses against these variants is likely in certain individuals. However, the high heterogeneity of T cell responses within vaccinated individuals, with an average individual targeting a median of 11 CD4+ and 10 CD8+ S-specific epitopes [39], makes widespread T cell escape in the population unlikely. Thus, T cell responses are expected to protect against severe disease from BA.2.86 and JN.1 infections, consistent with a recent clinical study conducted on JN.1 infected patients in a French cohort [41]. T cell recognition of BA.2.86 and JN.1 is also expected to be enhanced with the XBB.1.5 BNT162b2 booster vaccine currently recommended by the US CDC. This may be further enhanced by responses against non-S proteins, including memory T cell responses resulting from prior infection.

Our assessment of SARS-CoV-2 T cell cross-reactivity against BA.2.86 and JN.1 is based on epitope conservation analysis. This method has proven to be reliable for assessing the cross-reactivity of T cell responses to SARS-CoV-2 as well as other viruses including SARS-CoV-1, dengue virus, and monkeypox virus [38,42,43,44], and the predictions have aligned closely with experimental studies [18,19,20,21,22,45]. Nonetheless, the experimental quantification of T cell cross-reactivity against BA.2.86 and JN.1 is still required. Encouragingly, while this paper was under review, preliminary experimental studies related to BA.2.86 have emerged [46,47] that provide support for our findings. These studies indicate the preservation of cross-reactivity in vaccine-induced T cell responses against the BA.2.86 variant within specific cohorts. 

In addition to mutations and deletions, insertions have also been reported in some SARS-CoV-2 variants. However, based on sequence data deposited in GISAID and from the information available at www.outbreak.info [15], BA.2.86 does not have any insertions of notable consequence. Even the S214:EPE insertion found in earlier Omicron lineages is not present in BA.2.86. The only notable insertion is S16:MPLF, which appears in ~10% of available BA.2.86 sequences. This insertion does not affect any known T cell epitope.

The immune escape potential of epitope mutants was assessed with the use of peptide–HLA binding prediction tools (netMHCIIpan4.1 and netMHCpan4.1). The accuracy of these tools in predicting peptide–HLA binding is generally high, as indicated by a median positive predictive value (PPV) greater than 0.74 [17]. For HLA-DR and specific HLA-C alleles, a slightly lower accuracy was reported, with PPV values ranging between 0.5 and 0.7. This can be attributed to the limited availability of training data for these alleles [16,17]. Importantly, the affected epitopes associated with these alleles represent only a small fraction of the total epitopes considered in our study, specifically 7 out of 86 HLA-C-restricted epitopes and 11 out of 125 HLA-DQ-restricted epitopes (see Supplementary Tables S3 and S4). Therefore, the limitations stemming from the lower accuracy of these specific alleles have minimal impact on the overall conclusions drawn in our research. 

There are multiple limitations of our work. First, it is possible that a mutation that does not affect the HLA binding of an epitope may still result in loss of epitope recognition through other mechanisms, such as the abrogation of T cell receptor binding with the peptide–HLA complex [48]. This effect is not captured by our current analysis. In addition, while deletions in T cell epitopes may not always lead to the abrogation of binding, deletions do appear to have an impact on binding in many cases [49,50]. While we adopted a conservative approach by treating any epitope with a deletion as being lost, targeted T cell assays are required to assess these predictions. Second, the individual immune response is complex and influenced by various factors, including previous natural infections, the variants involved in those infections, homologous or heterologous vaccinations, the number of vaccine doses, time since the last dose or last infection, and other factors. However, due to the limited availability of information regarding T cell epitopes in these contexts, such factors could not be incorporated into the analysis. Third, our study of the putative robustness of T cell responses elicited by the XBB.1.5 booster against BA.2.86 is limited by a lack of epitope-specific data for the XBB.1.5 BNT162b2 booster. For this analysis, we were restricted to considering epitope sets that had sequence homology in both ancestral S and XBB.1.5 S proteins; hence, this excluded T cell epitopes that are specific to XBB.1.5 and do not share sequence homology to the ancestral S. Experimental epitope mapping is still required to further elucidate the set of T cell epitopes specific to the XBB.1.5 S antigen.

## Data Availability

The authors confirm that the data supporting the findings of this study are available within the article or in cited public databases.

## Supplementary Materials

The following supporting information can be downloaded at https://www.mdpi.com/article/10.3390/v16030473/s1, Figure S1: Overview of SARS-CoV-2 T cell epitopes across SARS-CoV-2 variants in proteins other than S. (A) and (C) show the fractions of CD4+ and CD8+ in non-S proteins of SARS-CoV-2 T cell epitopes with and without mutations across SARS-CoV-2 variants, along with breakdowns of BA.2.86 epitopes for which an HLA association is known. (B) and (D) show the predicted effect of Omicron BA.2.86 mutations on peptide–HLA binding. Table S1: List of CD4+ T cell epitopes affected by mutations in present in BA.2.86 and JN.1 variants. Table S2: List of CD8+ T cell epitopes affected by mutations in present in BA.2.86 and JN.1 variants. Table S3: List of CD4+ T cell epitope–HLA pairs affected by BA.2.86 and JN.1 mutations and their predicted effect on HLA binding. Table S4: List of CD8+ T cell epitope–HLA pairs affected by BA.2.86 and JN.1 mutations and their predicted effect on HLA binding.
