# Supplementary figures and images for: Cross-Reactivity Assessment of Vaccine-Derived SARS-CoV-2 T Cell Responses against BA.2.86 and JN.1
Source: Viruses. 2024 Mar 20;16(3):473. doi: 10.3390/v16030473 (PMC10975570; doi:10.3390/v16030473)

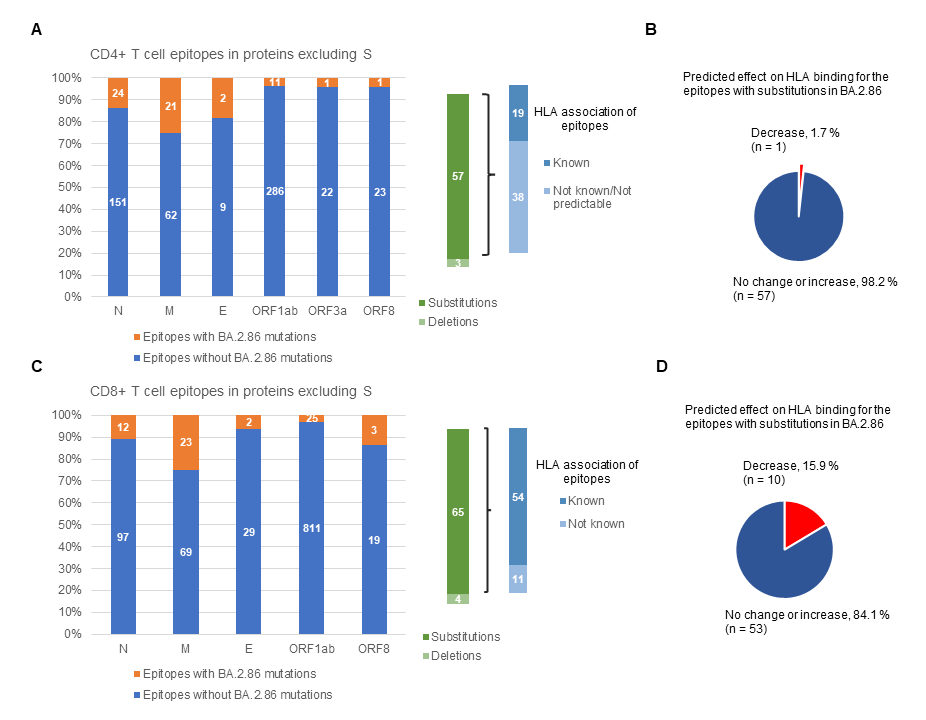

Supplement: Supplementary file 1 [file viruses-16-00473-s001.zip › Supp Fig.png]
